# B7H3-dependent myeloid-derived suppressor cell recruitment and activation in pulmonary fibrosis

**DOI:** 10.3389/fimmu.2022.901349

**Published:** 2022-08-15

**Authors:** Tianju Liu, Francina Gonzalez De Los Santos, Andrew E. Rinke, Chuling Fang, Kevin R. Flaherty, Sem H. Phan

**Affiliations:** ^1^ Departments of Pathology, University of Michigan Medical School, Ann Arbor, MI, United States; ^2^ Division of Pulmonary/Critical Care Medicine, University of Michigan Medical School, Ann Arbor, MI, United States

**Keywords:** B7H3, TERT, lung, fibrosis, myeloid-derived suppressor cell (MDSC)

## Abstract

Idiopathic pulmonary fibrosis (IPF) is a progressive fibrotic lung disease without effective curative therapy. Recent evidence shows increased circulating myeloid-derived suppressor cells (MDSCs) in cancer, inflammation, and fibrosis, with some of these cells expressing B7H3. We sought to investigate the role of MDSCs in IPF and its potential mediation *via* B7H3. Here we prospectively collected peripheral blood samples from IPF patients to analyze for circulating MDSCs and B7H3 expression to assess their clinical significance and potential impact on co-cultured lung fibroblasts and T-cell activation. In parallel, we assess MDSC recruitment and potential B7H3 dependence in a mouse model of pulmonary fibrosis. Expansion of MDSCs in IPF patients correlated with disease severity. Co-culture of soluble B7H3 (sB7H3)-treated mouse monocytic MDSCs (M-MDSCs), but not granulocytic MDSCs (G-MDSCs), activated lung fibroblasts and myofibroblast differentiation. Additionally, sB7H3 significantly enhanced MDSC suppression of T-cell proliferation. Activated M-MDSCs displayed elevated TGFβ and Arg1 expression relative to that in G-MDSCs. Treatment with anti-B7H3 antibodies inhibited bone marrow-derived MDSC recruitment into the bleomycin-injured lung, accompanied by reduced expression of inflammation and fibrosis markers. Selective telomerase reverse transcriptase (TERT) deficiency in myeloid cells also diminished MDSC recruitment associated with the reduced plasma level of sB7H3, lung recruitment of c-Kit^+^ hematopoietic progenitors, myofibroblast differentiation, and fibrosis. Lung single-cell RNA sequencing (scRNA-seq) revealed fibroblasts as a predominant potential source of sB7H3, and indeed the conditioned medium from activated mouse lung fibroblasts had a chemotactic effect on bone marrow (BM)-MDSC, which was abolished by B7H3 blocking antibody. Thus, in addition to their immunosuppressive activity, TERT and B7H3-dependent MDSC expansion/recruitment from BM could play a paracrine role to activate myofibroblast differentiation during pulmonary fibrosis with potential significance for disease progression mediated by sB7H3.

## Introduction

Fibroproliferative interstitial lung disease (ILD), such as idiopathic pulmonary fibrosis (IPF), is a chronic progressive disease often resulting in end-stage disease with a fatal outcome (1). It is characterized by mesenchymal cell recruitment, proliferation, and activation with *de novo* emergence and persistence of myofibroblasts (2, 3). Accumulating evidence suggests the importance of bone marrow (BM)-derived cells in fibrotic lung disease (4–9). Recruited hematopoietic progenitor cells (HPCs), HPC-derived innate lymphoid cells, and Ly6C^+^ monocytic cells promote lung fibrosis upon activation probably *via* paracrine mechanisms (6–9). BM-derived Ly6C^+^ cell recruitment into injured lung exacerbates bleomycin (BLM)-induced pulmonary fibrosis in a B7H3-dependent manner (8, 10).

In addition to its well-known role in host defense, myeloid cells are important in tissue remodeling (11). Of recent interest is myeloid-derived suppressor cells (MDSCs), which can negatively regulate immune responses in various diseases (11). MDSCs consist of two distinct subpopulations, monocytic MDSC (M-MDSC) and granulocytic MDSC (G-MDSC), as defined by certain cell surface markers (11, 12). In humans, these immunosuppressive MDSCs are characterized by the expression of CD33 and CD11b but lack markers of mature myeloid and lymphoid cells and the MHC class II molecule HLA-DR (CD33^+^CD11b^+^HLA-DR^−/lo^) (13–16). M-MDSC and G-MDSC are distinguished by CD14 and CD15 expression, respectively. Mouse M-MDSC is phenotypically CD11b^+^Ly6G^−^Ly6C^hi^ with low side scatter, whereas G-MDSC is CD11b^+^Ly6G^+^Ly6C^−/lo^ with high side scatter (16, 17). Expansion of MDSCs in peripheral blood is noted in certain conditions, including cancer (18, 19), chronic infection, inflammation, tissue damage, and remodeling (20–22). Notably, some of these conditions correlated with type 2 immune responses (21, 23). In cancer, MDSCs exert direct immunosuppressive functions against T and NK cells, while simultaneously recruiting T regulatory cells to further enhance immunosuppression. Moreover, they promote tumor invasion and metastasis through their purported angiogenic activities (16). Similarly increased circulating MDSCs occur in pulmonary hypertension and IPF (24, 25), which correlates with disease progression. However, the mechanism and significance of MDSC recruitment in lung injury and fibrosis remain unclear.

In this study, we presented evidence of increased circulating and lung MDSC recruitment mediated by B7H3, which could play significant paracrine roles in lung inflammation and fibrosis by promoting myofibroblast differentiation and an as-yet-unknown role associated with MDSC immunosuppressive capacity. Moreover, this novel finding has implications for the immunosuppressive role of B7H3 in cancer as mediated by MDSCs.

## Materials and methods

### Human subjects and peripheral blood cell isolations

Peripheral blood samples were obtained from 69 patients with IPF and five normal controls recruited from the University of Michigan Medical School hospital. All IPF patients had a confirmed diagnosis and met the criteria for the diagnosis as established by the American Thoracic Society (ATS) and the European Respiratory Society (ERS) (26). The patient demographics are shown in **Table 1** (more detailed clinical data are provided in the **Supplementary Material**). Human peripheral whole blood cells were collected after red blood cell lysis using red blood cell (RBC) lysis buffer (Cat# 00-4300-54, eBioscience Inc., San Diego, CA, USA). Peripheral blood mononuclear cells (PBMCs) were isolated by using BD Vacutainer™ mononuclear cell preparation (CPT) tubes (Cat# 02-685-125, Thermo Fisher Scientific, Waltham, MA, USA). Freshly isolated cells were resuspended in flow cytometry staining buffer (fluorescence-activated cell sorting (FACS), 1% bovine serum albumin (BSA) in phosphate-buffered saline (PBS) buffer) and then used for flow cytometry analysis on the same day for both whole blood cells and PBMCs.

**Table 1:** Demographics of clinical subjects.

Subject type	N	Agemean (range)	Sex	Ethnicity	Treatment	DLCO, % predicted
IPF	69	65 (43–89)	F: 21	Non-Hispanic: 68 Others: 1	Yes: 47	57.04 ± 15.4
			M: 49		No: 22	
Normal Control	5	60 (50–71)	F: 4	Non-Hispanic: 3 Others: 2		
			M: 1			

DLCO, diffusing capacity of the lungs for carbon monoxide; IPF, idiopathic pulmonary fibrosis.

### Animal model studies

Floxed telomerase reverse transcriptase (*Tert* ^fl/fl^) on a C57BL/6J background was generated as previously described (27). To generate a myeloid cell-specific *Tert* knockout (KO) mouse, the floxed *Tert* mice were crossed with LysM-Cre mice (B6.129P2-*Lyz2^tm1(cre)Ifo^*/J, stock #004781, Jackson Laboratory, Bar Harbor, ME, USA) that were backcrossed to C57BL/6 mice for at least six generations. The genotype of *Tert* ^fl/fl^, Cre^−/+^ mice was confirmed by PCR (data not shown), and the resulting LysM-expressing myeloid-specific *Tert* KO mice were used for the BLM model studies. Pulmonary fibrosis was induced in these *Tert* KO or relevant control LysM-Cre^+/−^ mice (referred to as wild type (WT) for simplicity) with BLM (Mead Johnson, NJ, USA) dissolved in sterile PBS by endotracheal instillation on day 0 at a dose of 2 units/kg body weight as before (28). Animals were randomly assigned to the indicated treatment groups (n = 5~10 mice in each group). The mice were euthanized on day 7 after BLM treatment, and the lungs were harvested rapidly for tissue RNA isolation and lung single cell isolation, or on day 21 for hydroxyproline assay and histopathological examination.

To assess the importance of B7H3 *in vivo*, blocking antibodies to mouse B7H3 (Bio X cell, Lebanon, NH, USA; Cat# BE0124; Clone# MJ18) or its isotype control rat IgG1 were injected into the mice i.v. *via* tail veins, every other day starting on day 1 after BLM treatment (0.3 mg/mouse). The mice were euthanized on day 7 after BLM treatment, and their lungs were harvested rapidly for RNA and single-cell isolation for flow cytometry analysis.

Mouse lung fibroblasts (MLFs) were isolated using a digestion cocktail containing collagenase III and DNase I (Worthington Biochemical Crop., Lakewood, NJ, USA) and maintained in Dulbecco’s modified Eagle’s medium (DMEM) supplemented with 10% plasma-derived fetal bovine serum (PDS; Animal Technologies, Tyler, TX, USA), 10 ng/ml of epidermal growth factor (EGF) and 5 ng/ml of platelet-derived growth factor (PDGF) (R&D Systems, Inc. Minneapolis, MN, USA) as before (29). The whole-lung single-cell suspension was prepared by digesting lung tissue using the same digestion cocktail and then resuspended in FACS buffer for flow cytometry analysis.

### Flow cytometry and cell sorting

For analysis of human circulating MDSCs, white blood cells and PBMCs were blocked first with anti-human FcγR (CD16/CD32) (clone: HP6017, Cat# 409302, BioLegend, San Diego, CA, USA) and then stained with a six−color cocktail of antibodies comprised of HLA-DR-Brilliant Violet (BV) 421 (clone L243, Cat# 307636), CD33-APCCy7 (clone P67.6, Cat# 366614), CD11b-BV 510 (clone M1/70, Cat# 101263), CD14-FITC (clone 63D3, Cat# 367116), CD15-PECy7 (clone HI98, Cat# 301906), CD84-PE (clone CD84.1.21, Cat# 326008), or B7H3-PE (clone DCN70, Cat# 331606). These antibodies were all purchased from BioLegend (San Diego, CA, USA). White blood cells were also stained for regulatory T cells using CD4-APC (clone A161A1, Cat# 357408, BioLegend), CD25-APCCy7 (clone BC96, Cat# 302614, BioLegend), and Foxp3-AF488 (clone MF-14, Cat# 126405, BioLegend). The data were then acquired with a NovoCyte flow cytometer and analyzed by NovoExpress software (Acea Biosciences, Inc., San Diego, CA, USA). MDSC gating strategy consisted of initial gating based on HLA-DR expression. The HLA-DR^−/lo^ population was then analyzed for the myeloid markers CD11b and CD33. Thus, the human total MDSCs were defined as HLA-DR^−/lo^CD11b^+^CD33^+^ cells comprised of the granulocytic subpopulation MDSC (G-MDSC) phenotypically further analyzed as CD15^+^CD14^−^, and the M-MDSC with a CD14^+^CD15^−^ phenotype. These MDSC subpopulations in peripheral blood samples were counted and shown as a percentage of the total number of leukocytes or PBMCs. Mouse whole-lung single-cell suspension was stained with BioLegend antibodies CD45-PECy7 (clone 30-F11, Cat# 103114), ckit-PE (clone 2B8, Cat# 105808), and lineage cocktail-APC (Cat# 558074, BD Biosciences, Franklin Lakes, NJ, USA) for HPCs. The cells were stained with CD11b-BV 510 (clone M1/70, Cat# 101263) and Gr1-BV421 (clone RB6-8C5, Cat# 108433) for identification and quantification of the MDSC population. Mouse MDSC population was gated as CD11b^+^Gr1^+^ cells and HPCs as lineage^−^CD45^+^ckit^+^ cells. Where indicated in soluble B7H3 (sB7H3)-treated BM cells, the cells were first gated by CD11b, and CD11b^+^ BM cells were then gated by CD84. G-MDSC or M-MDSC cells were analyzed in these CD84^+^ populations. The data were acquired and analyzed as described above for human cells. For mouse cell sorting, whole-lung single-cell suspension was stained as indicated by the following antibodies from BioLegend: CD45-PECy7, CD11b-BV510, Ly6C-FITC (clone HK1.4, Cat# 128006), and Ly6G-BV421 (clone 1A8, Cat# 127628). Lung CD45^+^, M-MDSC (CD11b^+^Ly6C^hi^Ly6G^−^), and G-MDSC (CD11b^+^Ly6C^-/lo^Ly6G^+^) cell sorting was performed using a Sony MA900 Cell Sorter (Sony Biotechnology, San Jose, CA, USA). For all human and mouse cell staining and sorting, the respective isotype controls conjugated with the same fluorochrome as their respective antibodies and each single-color antibody were included in the antibody mixes. Fluorescence minus one (FMO) controls were used for the rare populations of B7H3-PE and ckit-PE. These controls were used for cell gating.

### Real-time quantitative RT-PCR

For quantitative mRNA analysis, total RNA was isolated from lung tissue, fibroblasts, or sorted MDSC. The primers and TaqMan probes of the following genes were purchased from Applied Biosystems (Carlsbad, CA, USA): mouse TERT (*Tert*), type I procollagen (*Col1a2*), α-SMA (*Acta2*), TGFβ (*Tgfb*), and 18S rRNA. For each qPCR assay, 100 ng of sample total RNA was used as a template, and 50 ng of 18S rRNA was used as an internal control to normalize the amount of input RNA. Results were expressed as 2^−ΔΔCT^ as previously described (30).

### Transwell co-culture

Primary isolated MLFs (at passages 2–5) were plated in the bottom chambers of 12-well transwell plates with a pore size of 0.4 µm (BD Biosciences) at a density of 1 × 10^5^ cells/well 24 h prior to co-culture. BM cells were treated with granulocyte-macrophage colony-stimulating factor (GM-CSF) (0.01 µg/ml) combined with sB7H3 (4 µg/ml) or stem cell factor (SCF) (0.1 µg/ml, R&D Systems) for 3 days and then subjected to cell sorting to isolate MDSCs. The sorted G-MDSC and M-MDSC (1 × 10^5^ cells/insert) were added to the upper inserts of the plates, and the cells were co-cultured for an additional 48 h. The MLFs in the bottom wells were harvested for RNA isolation. A small portion of G-MDSCs and M-MDSCs not used in the co-culture were also collected for RNA isolation.

### Cell migration assay

Cell migration assays were performed using FluoroBlok™ 96-Multiwell Insert Plates with 3.0 µm High Density PET Membrane (#351161, Thermo Fisher Scientific, Waltham, MA, USA) as described before (10). The CD11b^+^Gr1^+^ MDSCs were isolated using the EasySep™ Mouse Myeloid-Derived Suppressor Cell Isolation kit (#19867, Stemcell Technologies, Cambridge, MA, USA) according to the manufacturer’s instructions. The isolated cells were prelabeled with 5 μM of calcein AM (#564061, BD Biosciences, San Jose, CA, USA) and then were seeded in the upper chamber at the density of 1 × 10^5^/50 µl/insert. Normal MLFs were cultured with or without (control) TGFβ treatment (10 ng/ml) for 24 h. The media containing residual TGFβ were removed, and after washing, fresh media were added. Conditioned media (CMs) were collected after an additional 48 h. The control or TGFβ-treated MLF CMs were incubated with B7H3 blocking antibody (15 µg/ml) or isotype IgG and then added to the lower chamber of the plates. The plates were incubated for 18 h followed by fluorescence measurement using SpectraMax Gemini EM Microplate Reader (Molecular Devices, San Jose, CA, USA).

### T-cell suppression assay

Freshly isolated mouse BM cells were treated with GM-CSF and sB7H3 as described above. The control cell was treated with GM-CSF only. Three days later, the CD11b^+^Gr1^+^ MDSCs were isolated/purified using the EasySep™ Mouse Myeloid-Derived Suppressor Cell Isolation kit (Stemcell Technologies) according to the manufacturer’s instructions. Splenocytes isolated from mouse spleen were pre-labeled with 5 µM of proliferation dye carboxyfluorescein succinimidyl ester (CFSE) (BD Biosciences) and then co-cultured with GM-CSF/sB7H3-activated MDSC (1:1 ratio) in the presence of CD3/28 Dynabeads (Life Technology, Thermo Fisher Scientific) and 50 U/ml of rmIL2 (R&D Systems) for 3 days. The cells were collected and stained with CD4-BV421 (BioLegend, Cat# 100437, clone GK1.5) and CD8-APC (BioLegend, Cat# 100711, clone 53-6.7). The intensity of incorporated CFSE in CD4^+^ and CD8^+^ populations was analyzed by flow cytometry as described above.

### Hydroxyproline assay and histopathological analysis

Whole-lung collagen content was evaluated by measuring the hydroxyproline content from the lung homogenates as described previously (31, 32). The results were expressed as µg HYP per lung.

For fibrosis examination, the lungs were inflated by intratracheal perfusion and fixed for 24 h with 10% buffered formaldehyde. Lung tissue was then paraffin-embedded, sectioned, and stained with hematoxylin and eosin (H&E).

### Soluble B7H3 ELISA

The levels of sB7H3 in mouse plasma samples were measured using an ELISA kit (LS BioSciences, Inc., Seattle, WA, USA), in accordance with the manufacturer’s protocol.

### Statistical analysis

All data were expressed as mean ± SD unless otherwise indicated. Differences between means of various treatment and control groups were assessed for statistical significance by analysis of ANOVA with Tukey’s multiple comparisons. Associations between variables were established by linear regression and Pearson’s correlation with a two-tailed p-value using GraphPad Prism (version 8.0.2, GraphPad Software, San Diego, CA, USA). p < 0.05 was considered to indicate statistical significance.

### Study approval

The human studies were reviewed and approved by the Institutional Review Board at the University of Michigan. Informed consent was obtained from each patient or normal volunteer. All animal studies were reviewed and approved by the Institutional Animal Care and Use Committee at the University of Michigan.

## Results

### Increased myeloid-derived suppressor cells in peripheral blood of patients with idiopathic pulmonary fibrosis

**Figure 1:**
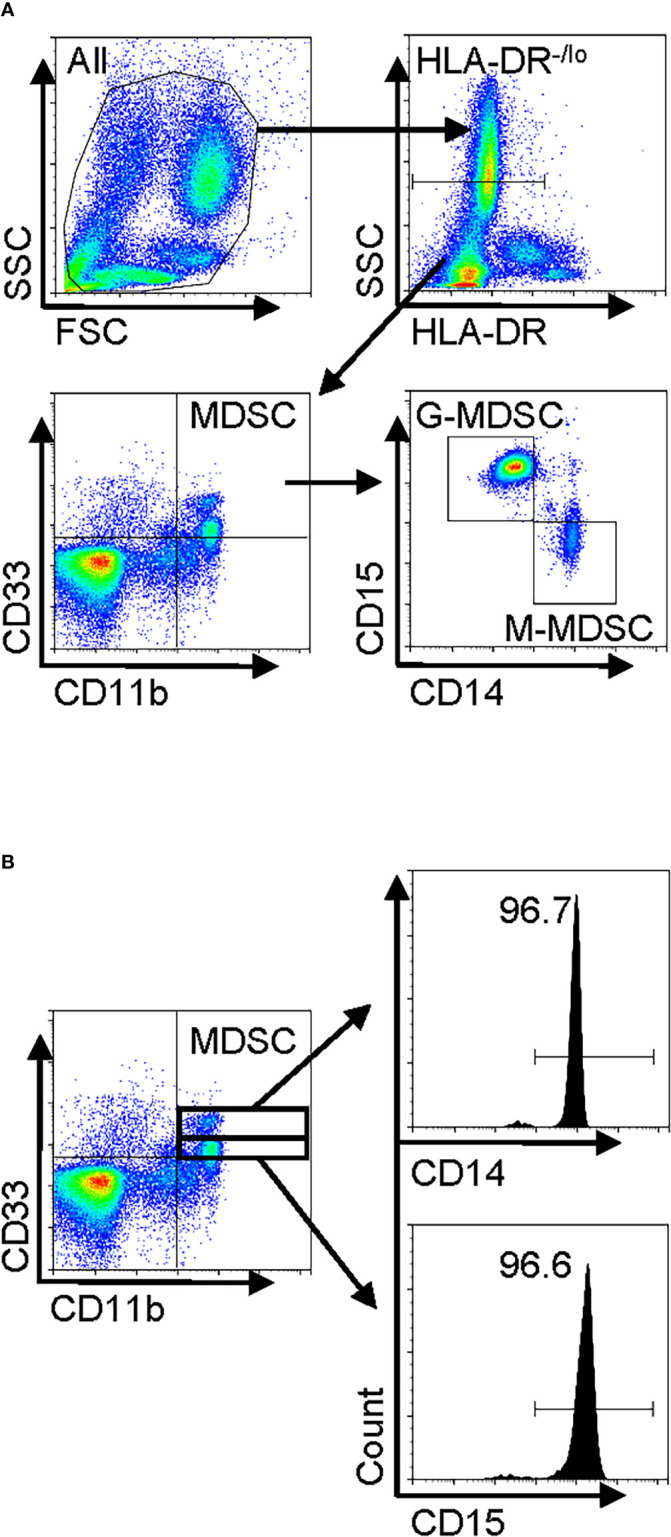
Human peripheral blood MDSC phenotype in IPF and gating strategy for flow cytometry. Freshly isolated whole blood leukocytes or PBMCs were stained with and analyzed by flow cytometry. **(A)** A representative set of flow cytometry gating strategy. The cells were first gated for HLA-DR^−/lo^, and then total MDSCs were defined from the HLA-DR^−/lo^ population as the CD11b^+^CD33^+^ fraction. The MDSC population was subsequently classified into two distinct subpopulations, namely, G-MDSCs (CD14^−^CD15^+^) and M-MDSCs (CD14^+^CD15^−^), by further analysis for CD14 and CD15 expression. **(B)** The MDSCs (HLA-DR^−/lo^CD11b^+^CD33^+^) were further gated for CD33^hi^ and CD33^lo^ followed by analysis for CD14 or CD15 expression. MDSC, myeloid-derived suppressor cell; IPF, idiopathic pulmonary fibrosis; PBMCs, peripheral blood mononuclear cells.

**Figure 2:**
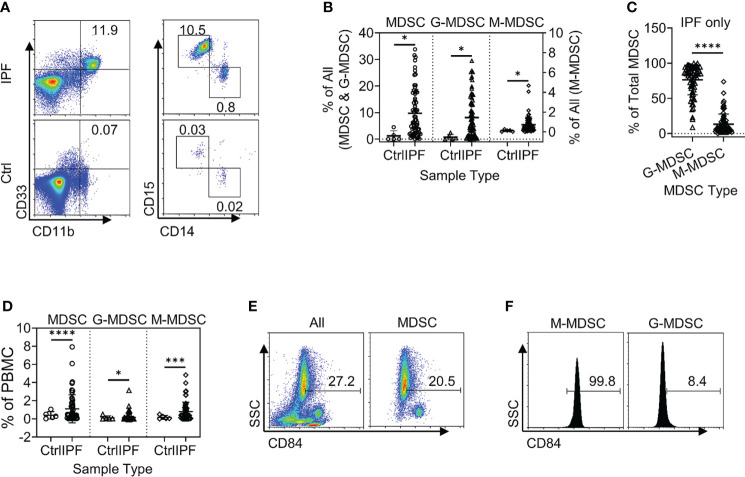
Circulating MDSCs were increased in the peripheral blood samples from patients with IPF. **(A)** A representative set of flow cytometry plots shows the total circulating MDSCs as well as G-MDSCs and M-MDSCs in IPF patient samples (top) *vs*. normal control (Ctrl, bottom). **(B)** The quantitative flow cytometry results of the frequencies for all three populations of MDSCs from the peripheral whole blood leukocyte samples of IPF patients are shown. The left y-axis indicates the frequencies of MDSCs and G-MDSCs, while the right y-axis indicates those for M-MDSCs. All values are shown as mean ± SD. N = 69 for IPF, and 5 for normal control. *p < 0.05 for all. **(C)** The distribution of the G-MDSC and M-MDSC subpopulations within the total MDSCs are shown as percentages of total MDSC. N = 69 (IPF samples only). ****p < 0.0001. **(D)** The quantitative results of the frequencies for the three populations of MDSCs in the PBMC fraction are shown. Mean ± SD with N = 56 for IPF, and 5 for normal control. ****p < 0.0001 for total MDSC, *p < 0.05 for G-MDSC, ***p < 0.001 for M-MDSC. **(E)** CD84^+^ cells are shown in whole blood leukocytes (left) and pre-gated MDSC population (right). **(F)** CD84^+^ cells are shown in pre-gated G-MDSC (left) and MDSC (right) subpopulations. Representative flow plots are shown in panels **(E, F)** MDSC, myeloid-derived suppressor cell; IPF, idiopathic pulmonary fibrosis; G-MDSCs, granulocytic myeloid-derived suppressor cells; M-MDSCs, monocytic myeloid-derived suppressor cells.

Total leukocytes and PBMCs isolated from freshly drawn peripheral blood samples were analyzed for MDSCs (defined as HLA-DR^−/lo^/CD33^+^/CD11b^+^ cells) and its two major subpopulations, G-MDSC (CD14^−^CD15^+^CD11b^+^CD33^+^HLA-DR^−/lo^) and M-MDSC (CD14^+^CD15^−^CD11b^+^CD33^+^HLA-DR^−/lo^) using the gating strategy shown in **Figure 1A**. Almost all CD33^hi^ MDSCs were CD14 positive, while the CD33^lo^ MDSCs were CD15 positive (**Figure 1B**). The percentages of all three MDSC populations were determined in both total leukocyte and PBMC fractions from the flow cytometry analysis. The results showed that the percentages of total MDSC (mean = 9.7%) and subpopulations G-MDSC (mean = 8.1%) and M-MDSC (mean = 0.73%) were all significantly and highly increased in samples from patients with IPF (p < 0.05 for all), relative to those from control subjects, wherein MDSCs were barely detectable (**Figures 2A, B**). There was a greater abundance (>10-fold on the average) of the G-MDSC subpopulation in the total leukocyte samples relative to M-MDSCs (76.4% *vs*. 13.2%), with greater patient-to-patient variability in the former *vs*. the latter (**Figure 2C**). Consequently, the markedly increased total MDSC in IPF patient samples was mostly due to the increase in G-MDSC. However, in the PBMC samples wherein the granulocytic fraction had been removed, the percentages of total MDSC (0.4 *vs*. 1.1%, p < 0.0001) and G-MDSCs (0.1 *vs*. 0.17, p < 0.05) were much lower relative than those before removal of granulocytes but remained higher in IPF *vs*. control samples (**Figure 2D**). In contrast, the frequencies of M-MDSC in PBMCs were essentially unchanged after the removal of granulocytes (0.17 *vs*. 0.81%, p < 0.001); thus, the level of circulating MDSCs was significantly elevated in the peripheral blood of IPF patients.

In further analysis of the MDSC subpopulations, we evaluated CD84 expression, which characterizes a novel MDSC subpopulation recently identified in breast cancer (33). Flow cytometric analysis of IPF patient blood samples revealed the presence of such a CD84^+^ population comprising 27.2% of total leukocytes, while 20.5% of the MDSC fraction was positive for CD84 (**Figure 2E**). Only ~8.4% of G-MDSCs were positive for CD84, and almost all M-MDSCs were uniformly CD84^+^ (**Figure 2F**). Thus, CD84 expression represented an additional phenotypic characteristic of M-MDSCs.

### Increased circulating myeloid-derived suppressor cells, Tregs, and lung function in idiopathic pulmonary fibrosis

**Figure 3:**
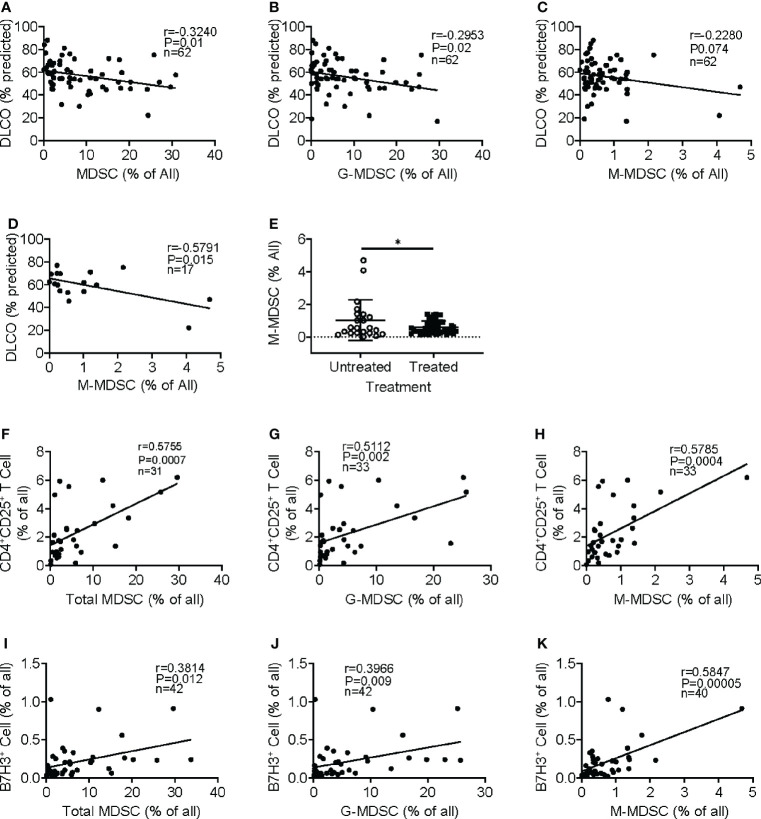
Clinical significance of circulating MDSCs and association with regulatory T cells and B7H3^+^ cells. Regression and correlation analyses were performed for MDSC abundance in peripheral blood and its relationship to lung function. The correlations between lung function (DLO, % predicted) and the frequencies of MDSCs **(A)**, G-MDSCs **(B)**, and M-MDSCs **(C)** are shown. N = 62. p < 0.05 in panels A–C. **(D)** The frequency of M-MDSCs in samples from the IPF patients without treatment was plotted for the correlation analysis. N = 17. p < 0.05. **(E)** The IPF patient samples were separated into untreated and treated (with pirfenidone or nintedanib) groups, and the M-MDSC frequency was compared between these two groups. N = 22 in untreated; N = 44 in treated IPF group. *p < 0.05. **(F)** Circulating CD4^+^CD25^+^ regulatory T cells were analyzed in whole blood showing a positive correlation between the frequency of whole blood total MDSC and regulatory T cells. N = 31. p < 0.001. CD4^+^CD25^+^ T-cell correlation between G-MDSC **(G)** and M-MDSC **(H)** is shown. N = 33. p < 0.001. The frequency of B7H3^+^ cells was analyzed, and the correlation with MDSC **(I)**, G-MDSC **(J)**, and M-MDSC **(K)** is shown. N = 42 in panels I and J, and 40 in panel **(K)** p < 0.05 in panel **(I)**, p < 0.01 in panel **(J)**, and p < 0.0001 in panel **(K)** MDSCs, myeloid-derived suppressor cells; DLCO, diffusing capacity of the lungs for carbon monoxide; G-MDSCs, granulocytic myeloid-derived suppressor cells; M-MDSCs, monocytic myeloid-derived suppressor cells.

We analyzed for any correlation of circulating MDSCs and Tregs with several lung function parameters, including the diffusing capacity of the lungs for carbon monoxide (DLCO), forced expiratory volume (FEV), and forced vital capacity (FVC). Initial evaluation revealed the absence of a significant association between MDSC abundance in the circulation and patients’ age, gender, FEV, or FVC (data not shown). However, the increased frequency of MDSC (**Figure 3A**) and G-MDSC (**Figure 3B**) in IPF blood samples significantly correlated with decreased DLCO, while M-MDSC abundance did not significantly (p-value = 0.07) correlate with DLCO (**Figure 3C**). Interestingly, upon segregation into treated (with pirfenidone or nintedanib or both) and untreated groups, only the M-MDSC abundance in IPF patients without clinical treatment was significantly negatively associated with DLCO (**Figure 3D**). Moreover, the M-MDSC frequency in this untreated group, but not that for total MDSC or G-MDSC, was significantly higher than that in treated patients. The difference became insignificant after the removal of the top two data points in the untreated group (**Figure 3E**). These results indicated a potential association between the abundance of MDSC and its subpopulations and the stage of disease progression and response to therapy. A larger number of cases may be needed to achieve statistical significance.

MDSCs play an immunosuppressive role in cancer and other chronic conditions (11, 18). Further analysis revealed that the emergence of MDCS in the total leukocyte samples was positively correlated with an increased number of circulating regulatory CD4^+^CD25^+^ T cells (**Figure 3F**). Similarly, both subpopulations of M-MDSC and G-MDSC exhibited positive correlations with CD4^+^CD25^+^ T cells (**Figures 3G, H**). Almost all CD25^+^ cells were positive for Foxp3, but only a small fraction was triple the CD4^+^CD25^+^Foxp3^+^ population within CD3^+^ T cells (data not shown). Similar results were obtained upon analysis of the PBMC samples (data not shown), suggesting a potentially immunosuppressive regulatory role for MDSC in IPF patients. Since B7H3 (CD276) is implicated in the mediation of expansion and migration of BM-derived MDSCs and IPF exacerbation (8, 10), we analyzed additionally for circulating B7H3^+^ cells in peripheral blood samples of IPF patients. The results showed that the increased number of B7H3^+^ cells was associated with the increased frequency of total MDSC in the total leukocyte samples (**Figure 3I**). The positive correlation with B7H3^+^ cells was also shown in both G-MDSC (**Figure 3J**) and M-MDSC subpopulations (**Figure 3K**), wherein M-MDSC accumulation showed a stronger correlation (r = 0.59) than G-MDSC (r = 0.39). These results indicated that B7H3-expressing cells were associated with the expansion of MDSC, especially with M-MDSC.

### Soluble B7H3 enhanced myeloid-derived suppressor cell promotion of lung fibroblast activation/myofibroblast differentiation and suppression of T-cell proliferation

**Figure 4:**
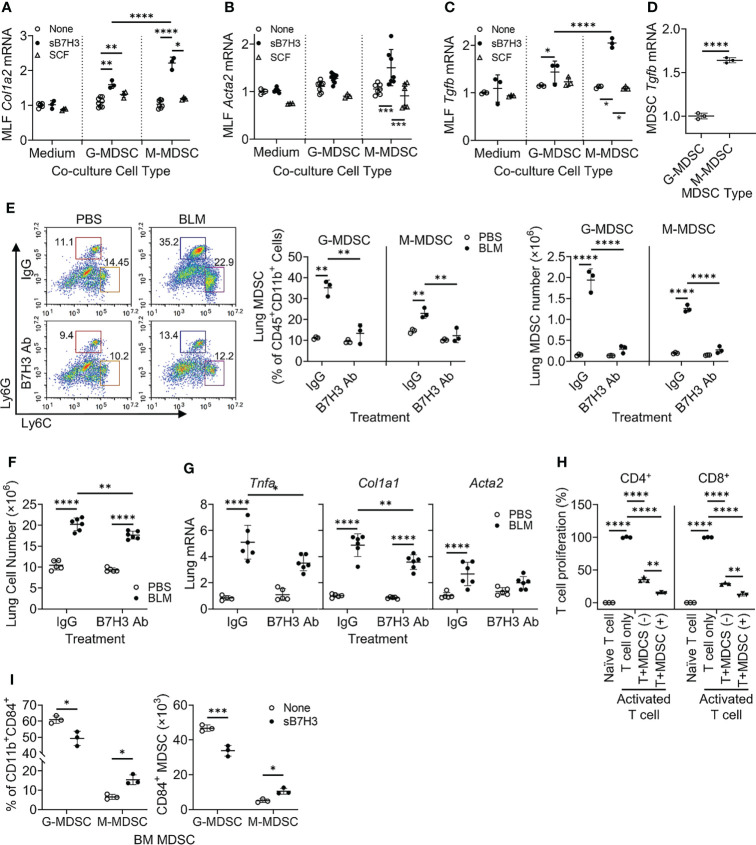
B7H3-activated MDSC promoted fibroblast activation/myofibroblast differentiation and suppressed T-cell proliferation. BM-derived MDSCs were treated with sB7H3 (4 µg/ml) or SCF (0.1 µg/ml) in presence of GM-CSF (0.01 µg/ml) for 3 days followed by flow cytometric cell sorting. Sorted G-MDSCs or M-MDSCs were co-cultured with primary isolated MLF in 24-well transwell plates (ratio of MDSC : MLF = 2:1). After 48 h of co-culture, RNA from MLF was isolated and analyzed by qPCR for type I collagen (Col1a2) **(A)**, α-SMA (Acta2) **(B)**, and TGFβ1 (Tgfb1) expression **(C)**. Cellular RNA from sorted G-MDSCs and M-MDSCs was also extracted and similarly analyzed for expression of TGFβ1 (Tgfb1) **(D)**. The 18S RNA was used as internal control for normalization. The data were expressed as fold change relative to the respective control. **(E)** B7H3 blocking antibody (Ab) was injected intravenously into mice after BLM treatment. The lung single-cell suspensions were obtained 7 days after BLM treatment for flow cytometry analysis of MDSCs. Representative plots (pre-gated by CD45^+^CD11b^+^ cells) are shown on the left panel and the quantitative analysis of percentages and absolute cell numbers on the middle and right panels, respectively. **(F)** Total lung cell numbers were counted using a hemocytometer. **(G)** Lung tissue RNA was analyzed by qPCR for *Tnfa*, *Col1a1*, and *Acta2* on day 7 after BLM treatment. **(H)** BM-derived CD11b^+^Gr1^+^ MDSCs with (+) or without (−) sB7H3 activation were co-cultured with CFSE pre-labeled splenocytes in media only (Naïve T cell) or in stimulation medium containing CD3/28 Dynabeads+rmIL2 (Activated T-cell) for 3 days. CD4^+^ or CD8^+^ T-cell proliferation was assessed by counting CFSE^+^ cells with either T-cell marker using flow cytometry. **(I)** Fresh BM cells were treated with sB7H3 for 72 h and analyzed for CD84 and MDSC markers. The data were shown as the percentage (left) or the absolute numbers per million BM cells (right) of G-MDSC or M-MDSC in the CD84-expressing CD11b^+^ BM population. Mean ± SD is shown for all. N = 3–8. *p < 0.05; **p < 0.01; ***p < 0.001; ****p < 0.0001. MDSC, myeloid-derived suppressor cell; BM, bone marrow; sB7H3, soluble B7H3; SCF, stem cell factor; GM-CSF, granulocyte-macrophage colony-stimulating factor; MLF, mouse lung fibroblast; BLM, bleomycin; CFSE, carboxyfluorescein succinimidyl ester.

The paracrine significance of B7H3 expression on MDSCs may be mediated by its membrane metalloproteinase-induced shedding as sB7H3 (34). To investigate this, MLFs were co-cultured with BM-derived G-MDSCs (CD11b^+^Ly6C^-/lo^Ly6G^+^) or M-MDSCs (CD11b^+^Ly6C^hi^Ly6G^−^) treated with either sB7H3 or SCF. Treatment with sB7H3 activated both G-MDSCs and M-MDSCs to significantly stimulate type I collagen expression in the co-cultured MLFs with a greater effect from M-MDSCs and only a modest effect from G-MDSCs (**Figure 4A**). Treatment with sB7H3 also activated M-MDSCs but not G-MDSCs to induce α-smooth muscle actin (α-SMA) expression in MLFs, indicative of myofibroblast differentiation (**Figure 4B**). Additionally, MLF TGFβ expression was induced by sB7H3-activated MDSCs, again with greater effect from M-MDSCs than G-MDSCs (**Figure 4C**). SCF had no significant effects on MDSC’s ability to induce either α-SMA or TGFβ in MLFs. The greater impact of sB7H3-treated M-MDSCs on co-cultured myofibroblast differentiation correlated with the significantly higher level of TGFβ expression relative to that in G-MDSCs (**Figure 4D**). These findings indicated that sB7H3-activated MDSC played a greater role in fibroblast activation and differentiation, a key event fibrosis formation, probably *via* paracrine release of TGFβ after activation.

To assess the *in vivo* importance of B7H3 in MDSC expansion during fibrosis, B7H3 blocking antibodies were injected (i.v.) into mice after BLM treatment. The results showed significantly increased CD45^+^ M-MDSCs and G-MDSCs in BLM-treated lungs, which were virtually abolished in animals treated with B7H3 blocking antibodies (**Figure 4E**). Total lung cell number was also decreased by this antibody treatment (**Figure 4F**), which was accompanied by a significant reduction in mRNA levels of TNFα, collagen I, and α-SMA (**Figure 4G**). Thus, B7H3 neutralization essentially eliminated BLM-induced BM-derived MDSC recruitment and a significant reduction in lung inflammatory and fibrotic responses.

To analyze the impact on MDSC immunosuppressive activity, mouse BM-derived CD11b^+^Gr1^+^ MDSCs without or with sB7H3 treatment were co-cultured with mouse splenocytes. The results showed that as expected, both CD4^+^ and CD8^+^ T-cells significantly proliferated in response to CD3/28 microbeads plus IL2 stimulation. Their proliferation was significantly suppressed by >60% in CD4^+^ and 70% in CD8^+^ T cells when co-cultured with MDSCs, which was significantly enhanced upon co-culture with sB7H3-treated MDSCs (**Figure 4H**). In addition to confirming MDSC immunosuppressive activity, these findings revealed a novel property of sB7H3 in the upregulation of this MDSC functionality. Since CD84 is a putative indicator of M-MDSC immunosuppressive activity (33), we evaluated the effect of sB7H3 on MDSC CD84 expression. Indeed sB7H3 treatment caused an increase in CD84-expressing M-MDSCs but a decline in the CD84-expressing G-MDSCs (**Figure 4I**). Thus, sB7H3-induced enrichment of the CD84^+^ M-MDSC population could potentially contribute to the enhanced immunosuppressive activity of M-MDSC.

**Figure 5:**
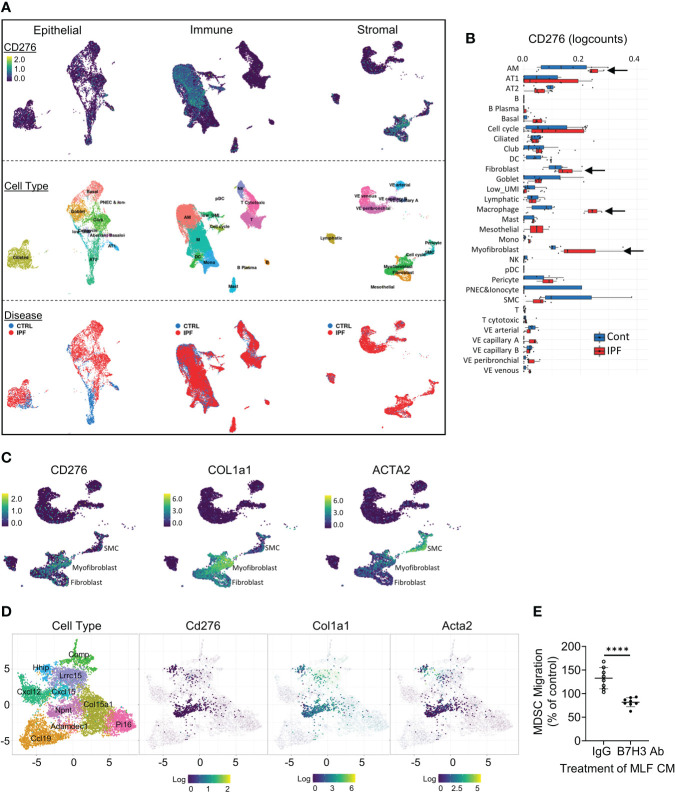
Lung scRNA-seq analysis identified B7H3-expressing cell clusters in human and mouse lung fibrosis. **(A)** Uniform manifold approximation and projection (UMAP) plots for all lung cells from IPF and control subjects were obtained from the IPF Cell Atlas online database (*CD276* expression was distributed in three distinct cell clusters shown in the top row). Diffusion map implementation labeled by cell type or disease status is shown in the middle row or the bottom row, respectively. **(B)** Level of *CD276* in the lung cell clusters in IPF *vs*. control subjects. **(C)** Distribution of the expression signals for the indicated genes within the stromal cell cluster. **(D)** UMAP plots (fibroXplorer.com) of mouse lung fibroblasts identified as Dpt+ universal fibroblasts from BLM-treated lungs. Gene expression level for each gene is shown in the fibroblast sub-clusters. Lung fibroblast sub-clusters are color-coded (see left panel). **(E)** Pre-labeled MDSCs isolated from naïve BM were placed in the upper inserts. Cell-free CMs collected from untreated (control CM) or TGFβ-treated MLF (TGFβ-CM) cultures were incubated with B7H3 blocking antibody (Ab) or control IgG prior to placing in the lower chambers. The fluorescence intensity of the lower chamber was measured at 18 h of incubation. The data are presented as the percentages of TGFβ CMs over their respective control CMs. Mean ± SD are shown. N = 8/group. ****p < 0.0001. scRNA-seq, single-cell RNA sequencing; IPF, idiopathic pulmonary fibrosis; BLM, bleomycin; CMs, conditioned media; MLF, mouse lung fibroblast.

### Cellular sources of B7H3 in fibrotic lung were revealed by single-cell RNA sequencing

To localize B7H3-expressing cells in injured lung, we reanalyzed single-cell RNA sequencing (scRNA-seq) data from the online database at IPF Cell Atlas (35) (GEO accession: GSE128033). In IPF, B7H3 gene (*CD276*)-expressing signals were observed mainly in fibroblasts/myofibroblasts, macrophages, and type I/II alveolar epithelial cells in the stromal, immune, and epithelial cell clusters, respectively (**Figure 5A**). Moreover, the expression level of *CD276* was elevated in these noteworthy cell clusters in IPF lung samples compared to those of control subjects (**Figure 5B**). Analysis of another scRNA-seq dataset showed a similar elevated expression pattern for *CD276* (36) (GEO accession: GSE134692). Interestingly, these analyses revealed a close association between the expression of *CD276* and myofibroblast differentiation markers, including α-SMA (*ACTA2*) and type I pro-collagen (*COL1A1*) in the fibroblast/myofibroblast clusters in the large stromal cluster (**Figure 5C**). Consistently, scRNA-seq analysis of transcriptomic signature of mouse lung fibroblasts using combined datasets from fibroXplorer.com (37) also revealed a close correlation among the expression signals of *Cd276*, *Col1a1*, and *Acta2*, mainly within three transcriptionally distinct sub-clusters identified as Npnt^+^, Hhip^+^, and Lrrc15^+^ fibroblasts (**Figure 5D**). These data identified the major cellular sources for induced B7H3 in the injured lung as being closely associated with differentiated myofibroblasts in both human and mouse lung fibrosis. Accordingly, we next sought to test this possibility by evaluating lung fibroblasts as a potential source of B7H3-dependent MDSC chemotactic activity, especially since B7H3 mRNA expression is elevated in fibroblasts isolated from the fibrotic lung or when treated with TGFβ (10). The results showed that TGFβ-treated MLF CMs significantly induced MDSC migration compared to the control CMs from untreated MLF, which was abolished by pre-incubation with neutralizing antibodies to B7H3 (**Figure 5E**). This finding is consistent with the lung myofibroblast as a source of B7H3 with chemotactic activity for MDSCs and their consequent recruitment to the lung.

### Myeloid TERT deficiency impaired myeloid-derived suppressor cell expansion/recruitment and reduced lung fibrosis

**Figure 6:**
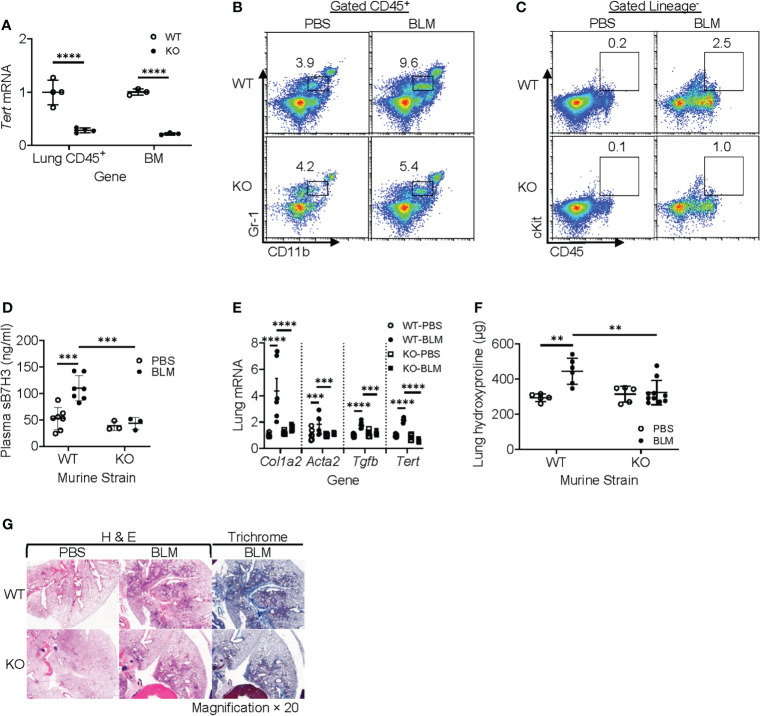
Myeloid-derived TERT deficiency abrogated recruitment of HPC and MDSC to injured lungs and reduced fibrosis. The lung injury/fibrosis model was induced in LysM-Cre/TERT KO and LysM-Cre WT control mice by BLM endotracheal injection. **(A)** BM cells and lung CD45^+^ cells were analyzed for TERT mRNA expression (*Tert*) by qPCR. 18S RNA was used as internal control for normalization. Mean ± SD are shown. N = 4. ****p < 0.0001. **(B)** Whole-lung single-cell suspension was analyzed for MDSCs (CD45^+^CD11b^+^Gr1^lo^) by flow cytometry and expressed as percentage of total lung cells. **(C)** Lung HPC (lineage^−^CD45^+^ckit^+^) was also analyzed by flow cytometry. The representative analyses from three separate flow cytometry runs (lung cells from three mice were pooled for each group per run) are shown. **(D)** Plasma level of sB7H3 was measured by ELISA. N = 7 in WT-PBS and WT-BLM, and 3 in KO-PBS and PBS-BLM. **(E)** Lung tissue RNA from WT or LysM-Cre/Tert KO mice was analyzed for type I collagen (*Col1a2*), α-SMA (*Acta2*), and TGFβ (*Tgfb)* mRNAs by qPCR. The data were expressed as fold change relative to the respective control. Mean ± SD are shown. N = 6 mice for all groups. ***p < 0.001 and ****p < 0.0001. **(F)** Lung tissues from WT or LysM-Cre/Tert KO mice were homogenized at day 21 after BLM or PBS treatment, and the lung collagen content was measured by hydroxyproline assay in whole-lung homogenates. Mean ± SD are shown. N = 5 mice in WT-PBS, 6 in WT-BLM, 5 in KO-PBS, and 10 in KO-BLM. **p < 0.01. **(G)** Representative H&E and Trichrome stained lung tissue sections are shown. Original magnification, ×20. TERT, telomerase reverse transcriptase; HPC, hematopoietic progenitor cell; MDSC, myeloid-derived suppressor cell; WT, wild type; BLM, bleomycin; PBS, phosphate-buffered saline.

TERT is a determinant for the proliferation and mobilization of HPCs (38–40), which are precursors of MDSCs (41, 42). To evaluate the role of myeloid cells and MDSCs *in vivo*, we generated a myeloid-specific LysM-Cre/Tert KO mouse line by crossing LysM-Cre with *floxed* Tert mice to explore the role of LysM-expressing myeloid cell-derived TERT on MDSC origination and expansion along with its impact on fibrosis. All WT and Cre/Tert KO mice survived up to day 21 after BLM treatment when the animals were euthanized for sample collection. The results confirmed that TERT expression was significantly ablated (~75% reduction) in BM and lung CD45^+^ cells of LysM-Cre/Tert KO as compared to WT mice (**Figure 6A**). BLM-induced lung injury caused a significant accumulation of MDSCs in WT mouse lungs (3.9 *vs*. 9.6% in PBS *vs*. BLM-treated mice), which was substantially reduced (4.2% *vs*. 5.4%) in myeloid cell TERT-deficient mice (**Figure 6B**). Furthermore, BLM-induced expansion of the lung ckit^+^-HPC (c-Kit^+^ CD45^+^lineage^−^) population observed in WT mice was also reduced in myeloid cell TERT-deficient mice (**Figure 6C**). Notably, this reduction in BLM-induced lung myeloid cell progenitor and MDSC expansion was accompanied by a significant reduction in plasma level of sB7H3 (**Figure 6D**).

Moreover, BLM-induced lung fibrosis in WT mice was significantly reduced in myeloid cell TERT-deficient mice as manifested by several markers of fibrosis, including α-SMA (*Acta2*), type I collagen (*col1a2*), and TGFβ (*Tgfb*) (**Figure 6E**). Consistently, the BLM-induced increase in lung hydroxyproline content was also essentially abrogated by TERT deficiency in the myeloid cell compartment (**Figure 6F**). The reduction in fibrosis was also manifested morphologically with smaller fibrotic lesions noted in the lungs of myeloid cell TERT-deficient mice (**Figure 6G**). These findings together suggested the potential importance of TERT in myeloid MDSC origination and expansion/accumulation in the injured/fibrotic lungs, likely by regulating HPC expansion and thus MDSC expansion/recruitment to the lung of consequence to subsequent fibrosis.

## Discussion

**Figure 7:**
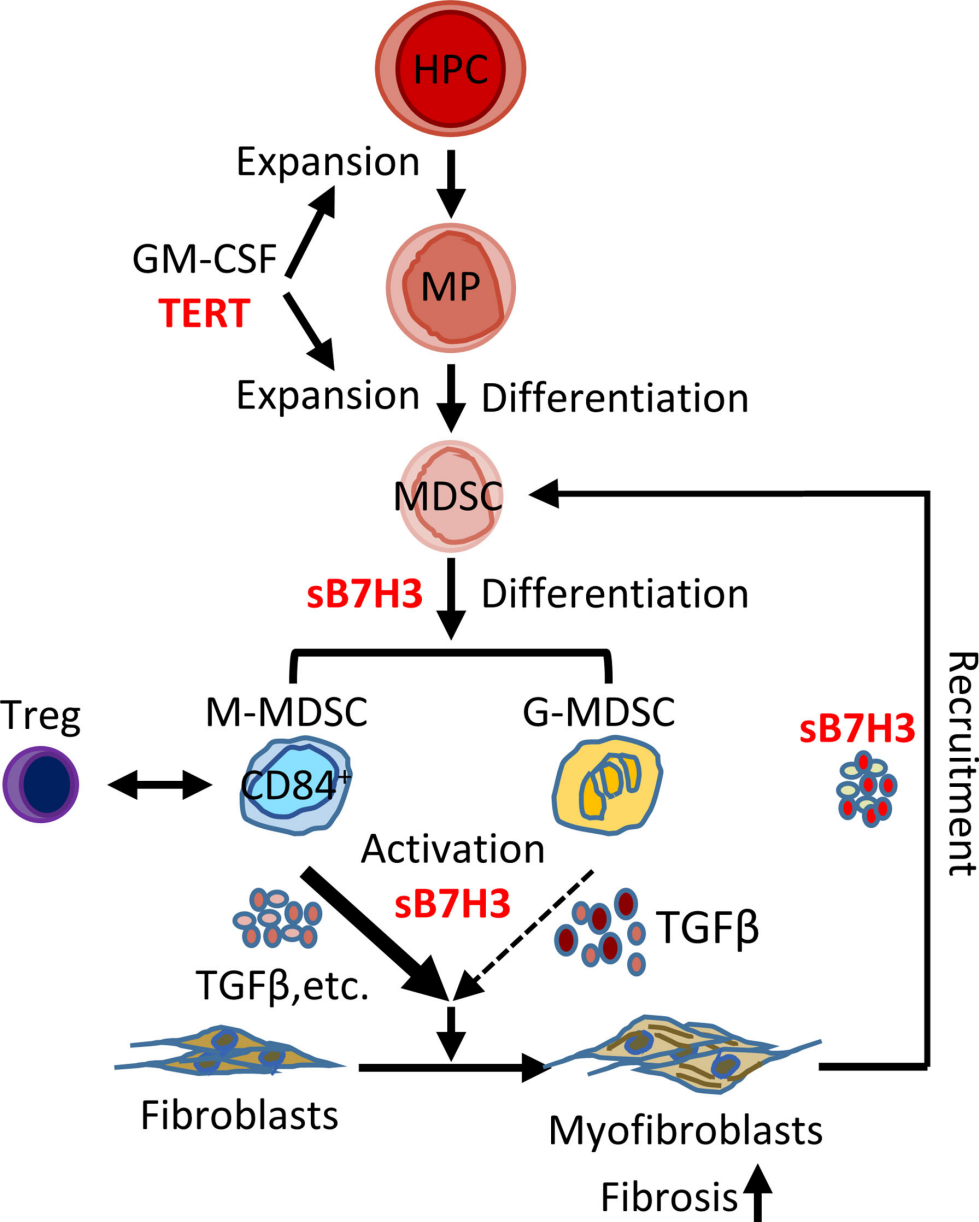
Schematic illustration of the proposed model for B7H3-dependent MDSC role in pulmonary fibrosis. In response to signals from injured lung, hematopoietic progenitor cells (HPCs) proliferate and give rise to myeloid precursors (MPs) under the control of TERT, GM-CSF, and other potential factors. TERT-expressing MP may govern the origination/expansion of MDSCs at intermediate stage of myeloid cell differentiation. Two subtypes of MDSC (G- and M-MDSC) were expanded and activated with the stimulation of GM-CSF/sB7H3 produced by injured lung tissue. The sB7H3-recruited and/or activated MDSCs, in turn, were able to induce resident lung fibroblast activation and/or myofibroblast differentiation through TGFβ production in a paracrine manner and with the greater impact of M-MDSC, thus promoting lung fibrosis. Lung scRNA-seq analysis-identified macrophages and fibroblasts/myofibroblasts were potential cellular sources for induced B7H3 in injured lungs. The findings suggested the potential importance of the observed elevated circulating MDSCs in IPF pathogenesis. In addition, elevated CD84^+^ suppressive cell-enriched M-MDSC showed some correlation with Tregs in peripheral blood of IPF patients, suggesting MDSC facilitation of immunosuppressive cell network in IPF as well. MDSC, myeloid-derived suppressor cell; TERT, telomerase reverse transcriptase; GM-CSF, granulocyte-macrophage colony-stimulating factor; G-MDSCs, granulocytic myeloid-derived suppressor cells; M-MDSCs, monocytic myeloid-derived suppressor cells; IPF, idiopathic pulmonary fibrosis.

Chronic fibrotic interstitial lung diseases such as IPF often rapidly progress to end-stage disease and fatal outcomes with limited treatment options (1, 43, 44). Recent evidence suggests that the expansion/accumulation of MDSCs may be involved in diverse pathogenic processes, including cancer, inflammation, and tissue injury, as well as IPF and pulmonary hypertension (11, 24, 25). Here we confirmed that circulating MDSCs were significantly elevated in the peripheral blood of patients with IPF, which correlated with diminished lung function and elevated circulating Tregs and B7H3-expressing cells. Further investigation revealed that sB7H3 could recruit/activate MDSCs to promote mouse lung fibroblast activation and differentiation in a paracrine manner potentially through TGFβ. Moreover, sB7H3 enhanced MDSC suppression of T-cell proliferation. *In vivo*, the importance of B7H3-dependent recruitment of MDSCs is revealed by evidence of diminished pulmonary fibrosis and myofibroblast differentiation in myeloid cell-specific TERT-deficient mice, which exhibited deficient lung MDSC expansion and recruitment in response to BLM-induced lung injury, along with a reduced level of plasma sB7H3. These findings suggested the potential importance of the observed elevated circulating MDSCs in IPF pathogenesis, with implications for their potential as a biomarker and/or therapeutic target in the management of IPF patients (**Figure 7**).

The MDSC population is highly heterogeneous and initially described as a loosely defined group of myeloid cells with immunosuppressive activity, especially in relation to T cells (11, 25, 33, 45, 46). The increase in MDSCs was associated with elevated circulating CD4^+^CD25^+^ regulatory T cells in IPF patient blood samples, especially with the elevation in M-MDSCs. Interestingly, virtually all of the M-MDSCs express CD84, thus resembling a novel suppressive CD84^+^ cell population identified recently in both human and murine MDSCs generated *in vitro* by GM-CSF treatment (33). CD84^hi^-MDSCs exhibit more robust T-cell suppression and reactive oxygen species (ROS) production compared with CD84^−/lo^ cells, which suggests that CD84 expression is a robust indicator of MDSC capacity (33), and consistent with the observation that M-MDSCs are more immunosuppressive than G-MDSCs in tumor-bearing mice (46, 47). Moreover sB7H3-activated MDSC enhanced suppression of T-cell proliferation, associated with enrichment of the CD84^+^ M-MDSC population. This might be relevant to the highly enriched CD84^+^ M-MDSC in IPF patient blood samples where the sB7H3 level was significantly increased. The significance of this MDSC immunosuppressive function in IPF is uncertain but likely to be due to the profibrogenic factors, such as TGFβ, also produced by these cells. This may be mediated by the elevated plasma level of sB7H3, since treatment with B7H3 blocking antibody eliminated BM-derived MDSC recruitment. In addition, consistent with our findings of sB7H3 ability to enhance MDSC immunosuppressive activity, a recent study shows that treatment with the same B7H3 antibody potently eliminates cancer stem cells and inhibits metastasis by enhancing CD8^+^ T cell-mediated antitumor immunity (48).

MDSCs appear to have multiple diverse roles in cancer, infection, and tissue injury and repair (20–22, 24, 49). MDSC numbers are positively correlated with clinical cancer stage, tumor burden, and poorer outcomes in various cancers, including colorectal carcinomas, breast, bladder, and non-small cell lung cancer (NSCLC) (11, 21, 50). We found that the increase of total MDSC and both subpopulations of G-MDSC and M-MDSC in the peripheral blood leukocytes of IPF patients correlated to a certain degree with a decline in lung function (DLCO) in patients with IPF. This is consistent with a previous study showing that MDSC levels inversely correlate with lung maximum vital capacity only in IPF, but not in non-IPF ILD. The proportion of M-MDSC, but not G-MDSC, is elevated in whole blood samples of IPF patients, while total MDSC accumulation is higher in IPF and non-IPF interstitial lung disease (25). In contrast, another study shows an increase in circulating CXCR2-expressing G-MDSCs but not M-MDSCs in IPF, as well as in mouse models of lung fibrosis and hypertension (24). Nevertheless, these findings together provide evidence that the expansion or accumulation of circulating MDSCs is a significant finding in IPF, despite the discrepancy in the relative increases of the MDSC subpopulations. The basis for the discrepancy is unclear but may be due to differences in the markers used for cell type identification, sample collection at different disease stages, or different therapies that patients received. Interestingly, the accumulation of circulating MDSC was also associated with an increased number of B7H3^+^ cells, which is suggested as a mediator for monocytic cell expansion and exacerbation of IPF (8, 10). In addition to expanded circulating MDSCs in IPF, CD33^+^CD11b^+^ cells are present in fibrotic lesions neighboring α-SMA-positive areas in the lung parenchyma (25). Consistent with this observation, our study also revealed that MDSC recruitment into fibrotic lung tissue was significantly increased upon BLM treatment. Together with both *in vitro* and *in vivo* findings of sB7H3-enhanced MDSC activation on myofibroblast differentiation, this evidence suggested the potential diverse roles of MDSCs in the promotion of fibrosis, in addition to their immunosuppressive function (24, 51). Together, these findings suggest that the abundance of MDSC and the level of plasma sB7H3 may represent useful prognostic biomarkers for disease severity and/or progression, and perhaps also as targets for therapeutic intervention to reduce or eliminate MDSC abundance.

The mechanism of MDSC expansion and activation in IPF is unclear. The findings suggested a potential role for sB7H3 perhaps in conjunction with other factors, such as GM-CSF or SCF released by injured lung, in their generation from BM cells. The clinical significance of sB7H3 was suggested by lung scRNA-seq analysis, which revealed type I/II alveolar epithelial cells, macrophages, and fibroblasts/myofibroblasts as the potential sources for sB7H3, with the latter as the predominant source. This possibility was supported by the identification of lung myofibroblast-derived sB7H3 as the key MDSC chemoattractant. Moreover, sB7H3 could expand/recruit the TGFβ-expressing MDSCs and in turn activate them to induce myofibroblast differentiation in a feedforward loop. The findings further supported the role of B7H3 in the recruitment of MDSCs, as well as enhancing the lung inflammation after injury in mice and the exacerbation of IPF in humans (8, 10). This expansion of MDSCs and accompanying fibrosis appeared to be dependent on TERT as shown by its abrogation in myeloid cell-specific (LysM expressing cells) TERT-deficient mice.

It is conceivable that a ‘two-signal’ model consisting of two processes might account for the expansion of MDSCs followed by their activation (42). The first process of MDSC expansion is induced by various cytokines produced by injured lung tissue, tumors, or bone marrow stroma in response to chronic stimulation, such as GM-CSF, M-CSF, G-CSF, IL-6, and VEGF. Our findings suggested that lack of TERT in myeloid cells diminished MDSC recruitment in response to BLM-induced lung insult. Given that TERT is implicated in cell proliferation and expansion of progenitor cells (28, 38, 52, 53), the evidence in the current study suggested that TERT may govern the origination or expansion of BM-derived MDSC during the first process of the ‘two-signal’ model. The capacity of TERT/telomerase to inhibit cell differentiation (54, 55) is in line with the observation that the signaling in the first process prevents differentiation of MDSC while promoting the proliferation of immature myeloid cells (11, 42). However, MDSCs may require a second signal to become activated, which manifests in the upregulation of immunosuppressive cytokine production. This stimulus for this activation can be provided by pro-inflammatory molecules such as IFNγ, IL-1β, IL-13, and TLR ligands (42, 56). The GM-CSF and sB7H3 combination served this dual purpose in our study with the secondary signal inducing TGFβ, which may in turn cause the myofibroblast differentiation.

In conclusion, CD84^+^ suppressive cell-enriched MDSCs are significantly elevated in peripheral blood of patients with IPF, which showed some correlation with lung function decline and evidence of T-cell suppression. These results suggested that MDSCs facilitate an immunosuppressive cell network in IPF. Moreover, the present study, for the first time, suggested that myeloid-derived TERT and sB7H3 might be important factors in the expansion of the circulating MDSC population, and its activation played a paracrine role by activating resident lung myofibroblast differentiation *via* the production of TGFβ, thus promoting lung fibrosis.

## Data availability statement

The raw data supporting the conclusions of this article will be made available by the authors, without undue reservation.

## Ethics statement

This study was reviewed and approved by the Institutional Review Board at the University of Michigan. Informed consent was obtained from each patient or normal volunteer. All animal studies were reviewed and approved by the Institutional Animal Care and Use Committee at the University of Michigan.

## Author contributions

SP and TL conceived the project. KF recruited the human subjects and provided patient demographics plus clinical advice. SP and TL designed the experiments. TL, FG, AR, and CF performed the experiments. SP and TL analyzed data and wrote the manuscript. All authors contributed to the article and approved the submitted version.

## Funding

This study is supported by NIH grants HL112880, HL138417, and HL143339.

## Acknowledgments

We thank Candace Flaherty and Christi Getty for their excellent assistance in the clinical blood specimen collection and thank Lisa R Riggs for excellent technical assistance in the tissue section preparation and the H&E and Masson’s trichrome staining.

## Conflict of interest

The authors declare that the research was conducted in the absence of any commercial or financial relationships that could be construed as a potential conflict of interest.

## Publisher’s note

All claims expressed in this article are solely those of the authors and do not necessarily represent those of their affiliated organizations, or those of the publisher, the editors and the reviewers. Any product that may be evaluated in this article, or claim that may be made by its manufacturer, is not guaranteed or endorsed by the publisher.
